# *Aeribacillus pallidus* Inoculant Orchestrates Functional Microbial Succession for Enhanced Nitrogen Transformation in High-Protein Waste Composting

**DOI:** 10.3390/microorganisms14030589

**Published:** 2026-03-05

**Authors:** Suhua Li, Ming J. Wu, Qinhong Yang, Jia Yang, Hongmin Yang, Zhiyong Zhao, Hongbin Yin

**Affiliations:** 1 College of Biological Science and Food Engineering, Southwest Forestry University, Kunming 650224, China; yang-qinhong@swfu.edu.cn (Q.Y.); yangjia0806@swfu.edu.cn (J.Y.); yanghongmin@swfu.edu.cn (H.Y.); 2 School of Science, Western Sydney University, Locked Bag 1797, Penrith, NSW 2751, Australia; m.wu@westernsydney.edu.au; 3 Yunnan Academy of Animal Husbandry and Veterinary Sciences, Kunming 650224, China; 4 Yunnan Animal Disease Prevention and Control Center, Kunming 650201, China

**Keywords:** *Aeribacillus pallidus*, meat and bone meal, nitrogen transformation, composting, bacterial community

## Abstract

The valorization of protein-rich meat and bone meal (MBM) via composting is hampered by significant nitrogen loss. Genomic analysis of *Aeribacillus pallidus* (*A. pallidus*) strain 60 revealed a genetic repertoire encoding potent proteolysis and nitrogen assimilation. We hypothesized that this strain could function as a microbial catalyst to redirect nitrogen flux during MBM composting. In a laboratory-scale trial, inoculation with *A. pallidus* triggered a rapid thermal surge (reaching 70 °C) and proteolytic cascade, significantly accelerating maturation. Crucially, this process enhanced relative nitrogen retention, increasing final total Kjeldahl nitrogen (TKN) concentration by 10.87–13.33% and nitrate by 13.75–18.65% compared to controls. Physicochemical and microbial profiling revealed that these improvements were driven by an inoculant-induced environmental modification rather than sustained inoculant dominance. The created thermal niche facilitated a distinct two-stage succession: an initial enrichment of proteolytic genera (*Thermoactinomyces*, *Ammoniibacillus*) followed by the establishment of a putative nitrifying community dominated by *Pseudoxanthomonas*. This study illustrates how a pioneer inoculant can drive functional microbiome assembly through niche modulation, providing a targeted strategy for optimizing nitrogen recovery in protein-dense waste valorization.

## 1. Introduction

The management of livestock mortality, particularly during epizootic outbreaks, presents a multifaceted global challenge [1]. Modern rendering systems ensure biosafety by converting animal carcasses into protein-rich meat and bone meal (MBM), a by-product containing approximately 8% total nitrogen [2]. Compositional analysis indicates that MBM is a challenging substrate, with over 80% of its protein locked in soft tissues and a significant fraction (17–26%) being resilient collagen [3]. This impedes the efficient utilization of MBM-derived nitrogen.

**Figure 1:**
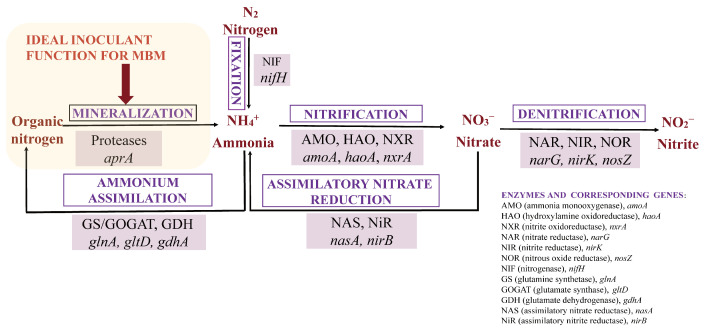
Conceptual framework of the nitrogen transformation network and bioaugmentation role in MBM composting. Metabolic pathways (mineralization, nitrification, denitrification, fixation) govern nitrogen flux, with corresponding enzymes and marker genes listed. Highlighted arrows indicate the hypothesized ideal inoculant function for MBM composting: accelerating proteolysis and mineralization to optimize nitrogen transformation.

With legislative restrictions limiting the use of MBM in animal feed, its valorization as an agricultural resource for organic fertilizer is paramount. Aerobic composting is a key technology for transforming MBM’s nitrogen into a stable organic fertilizer. The underlying biological network comprises four competing pathways: organic nitrogen mineralization, nitrification, denitrification, and nitrogen fixation, all of which interact with the crucial pathway of microbial assimilation [4,5]. The specific enzymatic steps and corresponding marker genes—such as ammonia monooxygenase (AMO, *amoA*) for nitrification and nitrate reductase (NAR, *narG*) for denitrification—for these pathways are visually summarized in the conceptual framework (Figure 1). Therefore, an ideal composting process for MBM must not only promote mineralization and nitrification but also maximize the flux of nitrogen into microbial assimilation, while actively suppressing denitrification and ammonia volatilization.

Currently, the direct application of MBM to agricultural land is common practice. However, without controlled stabilization, this approach leads to significant nitrogen losses (10–30%) through ammonia volatilization and denitrification, contributing to greenhouse gas emissions [6]. To mitigate these inefficiencies, bioaugmentation has emerged as a promising strategy. By introducing specific microbial inoculants, this approach aims to optimize the succession of the native microbial community and steer the metabolic networks toward conservation [7,8].

Despite its potential, current bioaugmentation research has predominantly focused on enhancing the degradation of recalcitrant lignocellulose in agricultural residues [9,10,11] or lipids in oily food waste [12]. Consequently, there is a paucity of specialized inoculants tailored for protein-dense substrates like MBM. For such materials, an effective inoculant must possess specific metabolic capabilities, primarily high proteolytic activity to initiate decomposition and thermotolerance to thrive in the composting environment.

*Aeribacillus pallidus* (*A. pallidus*), a species known for its remarkable thermostability and potent biodegradative capabilities, has been isolated from diverse, harsh environments [7,13,14,15,16]. Building on this foundation, our research group previously isolated strain 60 from rendered MBM, confirmed its identity as *A. pallidus* through 16S rRNA gene analysis, and characterized its high capacity for extracellular protease production [17]. This background identifies strain 60 as a prime candidate to initiate the challenging process of MBM decomposition. Therefore, we hypothesized that by leveraging its high proteolytic activity, inoculation with *A. pallidus* strain 60 would alter the balance of the microbial nitrogen transformation network, leading to improved overall nitrogen conservation in the final compost. In this study, nitrogen conservation was operationally defined as the maximization of Total Kjeldahl Nitrogen (TKN) retention, coupled with the efficient transformation of mineralized nitrogen into stable pools, specifically nitrate and humic substances. To test this hypothesis, we performed a comprehensive composting trial tracking physicochemical changes, key enzymatic activities, and microbial community succession, complemented by whole-genome sequencing of strain 60 to provide a genomic perspective on its observed performance.

## 2. Materials and Methods

### 2.1. Composting Materials and Microbial Inoculants

MBM derived from rendered swine carcasses was supplied by Yunnan Changteng Agriculture and Animal Husbandry Technology Co., Ltd. (Baoshan, China). Wheat straw was collected from agricultural areas near Kunming, China, air-dried, and milled to ≤3 cm fragments. Key physicochemical properties of raw materials are presented in Table 1. The thermophilic *A. pallidus* strain 60 (deposited in the China Center for Type Culture Collection under the accession number CCTCC M 20251328), previously isolated from composting MBM and characterized for its high protease production [17], was from our laboratory culture collection. A commercial organic decomposer was obtained from Henan Wobao Biotechnology Co., Ltd. (Hebi, China), containing *Bacillus subtilis* and *Aspergillus oryzae*.

### 2.2. Experimental Design and Sampling

The composting feedstock was prepared by homogenizing MBM and wheat straw at a 25:1 carbon-to-nitrogen ratio (C/N, dry weight basis), with initial moisture content adjusted to 65% (*w*/*w*) using deionized water. Three experimental treatments were established: (1) inoculation with *A. pallidus* strain 60 (A_60), (2) amendment with the commercial organic decomposer (COM), and (3) a sterile control (CON). Each treatment consisted of three independent conical piles (1.5 m base diameter, 0.8 m height) serving as biological replicates (*n* = 3).

To ensure strict comparability for nutrient addition, sterile Luria–Bertani (LB) broth was utilized as the carrier medium for all three treatments. For the A_60 treatment, *A. pallidus* strain 60 was cultured in LB broth at 55 °C for 24 h with continuous shaking at 180 rpm to reach the exponential phase. Cells were harvested by centrifugation (8000 rpm, 10 min), washed, and resuspended in fresh sterile LB broth. The final cell density was standardized to approximately 1.0 × 10^8^ CFU mL^−1^, by adjusting the turbidity to match a 0.5 McFarland standard, confirmed by optical density (OD_600_). For the COM treatment, the commercial decomposer powder was dissolved directly into sterile LB broth to achieve an equivalent concentration (1.0 × 10^8^ CFU mL^−1^) based on the manufacturer’s stated total viable count. The CON treatment consisted of an equivalent volume of sterile LB broth.

On day 0, inoculation was performed by evenly spraying 100 mL of the respective liquid suspension onto the composting materials while manually turning the piles to ensure homogeneous distribution. This application provided a theoretical initial inoculation density of 1.0 × 10^8^ CFU kg^−1^ dry weight in the biological treatments. No further inoculation was performed during the composting process.

During the 50-day composting period, temperatures were monitored three times daily (09:00, 14:00, 19:00) at pile centers and ambient locations. Moisture content was maintained at 50–65% (*w*/*w*) through periodic replenishment of deionized water, and manual turning was performed every 72 h to ensure aerobic conditions. Temporal samples (composting days 0, 1, 3, 6, 10, 15, 22, 30, 37, and 50) were collected from five spatially distributed points within each pile: the center, two radial points at mid-height (~40 cm from the base), and two points near the surface (~10 cm depth) (Supplementary Figure S1) [18]. The five subsamples from each pile were thoroughly mixed to form a single composite sample per pile per time point. Samples were homogenized, partitioned and stored at 4 °C for physicochemical analysis or at −80 °C for molecular enzymatic studies.

### 2.3. Physicochemical Analyses

All physicochemical analyses were performed on the composite samples collected from the three independent biological replicates (*n* = 3) following the national standards (NY/T 1867, NY/T 1116, and NY/T 525) of the People’s Republic of China. All chemicals were of analytical grade unless otherwise specified.

Sample aqueous extracts were prepared at a solid-to-water ratio of 1:10 (*w*/*v*) using deionized water. The mixtures were shaken at 200 rpm for 30 min at room temperature, followed by sedimentation for 30 min. The resulting supernatant was used to measure pH with a calibrated pH meter (PHSJ-3F, Shanghai INESA Scientific Instrument Co., Ltd., Shanghai, China) and electrical conductivity (EC) with an EC meter (DDSJ-319L, Shanghai INESA Scientific Instrument Co., Ltd., Shanghai, China).

Total organic carbon (TOC) was determined using the potassium dichromate oxidation-volumetry method. Briefly, samples were digested with 0.4 M K_2_Cr_2_O_7_ and concentrated H_2_SO_4_ (1:1 *v*/*v*) at 170–180 °C for 5 min. The residual oxidant was titrated with a standardized FeSO_4_ solution (approx. 0.2 M) using a phenanthroline indicator (endpoint: deep green to brick red), with blank correction applied. TOC content was calculated based on the consumption of the titrant using the following Equation (1):

(1) $$TOC(\%)\;=\;\frac{\left(V_{0}-\;V\right)\;\times C\;\times 0.003}{m}\;\times 100$$

where *V*_0_ and *V* are the titration volumes of the blank and sample (mL), *C* is the precise molarity of the FeSO_4_ solution determined by standardization against a K_2_Cr_2_O_7_ reference (mol L^−1^), 0.003 is the milliequivalent weight of carbon (g) corresponding to 1 mL of 1 M FeSO_4_, and *m* is the sample weight (g).

Humic substances were extracted with a mixture of 0.1 M Na_4_P_2_O_7_ and 0.1 M NaOH at a solid-to-liquid ratio of 1:20 (*w*/*v*). The suspension was shaken for 30 min at room temperature and centrifuged to obtain the supernatant. The total content of humic acid (HA) and fulvic acid (FA) in the supernatant was quantified using the same potassium dichromate oxidation method described above. To isolate HA, an aliquot of the supernatant was acidified to pH 1.5 using 6 M HCl to precipitate the HA fraction. The precipitate was separated by centrifugation, washed, and re-dissolved in 0.1 M NaHCO_3_ for carbon determination. The FA content was calculated as the difference between total humic substances (HA + FA) and HA. The humification index was calculated as the ratio of HA to FA (HA/FA).

Nitrogen fractions were quantified using an automated Kjeldahl analyzer (Kjeltec™ 8400, Foss A/S, Hillerød, Denmark). Total Kjeldahl nitrogen (TKN) was determined by digesting samples with concentrated H_2_SO_4_ and a catalyst mixture (CuSO_4_: K_2_SO_4_, 1:10 *w*/*w*) at 380 °C until a clear blue-green solution was obtained. The digestate was distilled with 40% NaOH, absorbed in 2% boric acid containing a mixed indicator (methyl red: bromocresol green, 1:5), and titrated against standardized 0.01 M HCl to a wine-red endpoint. TKN content was calculated based on the acid consumption (1 mL 0.01 M HCl ≈ 0.14 mg N). Ammonium nitrogen (NH_4_^+^-N) was determined by direct distillation of fresh compost samples with MgO under weak alkaline conditions. Nitrate nitrogen (NO_3_^−^-N) was extracted with 1 M HCl (solid-to-liquid ratio of 1:10). To eliminate interference from dissolved organic matter (DOM) and turbidity, the extracts were pre-treated by adding activated carbon for decolorization, followed by dry filtration to obtain a clear, colorless supernatant. The absorbance of the clarified filtrate was measured at 210 nm using a UV-vis spectrophotometer (UV-1800, Mapada Instruments Co., Ltd., Shanghai, China). The final nitrate concentration was calculated based on a standard curve generated using KNO_3_ standards.

### 2.4. Analysis of Nitrogen Cycling Functions by Enzyme Activities and Gene Abundances

To investigate the functional potential and activity of the microbial community, we quantified key enzymes and genes associated with four major nitrogen cycling pathways: organic nitrogen mineralization (protease; *aprA*), nitrification (AMO; *amoA*, *nxrA*), denitrification (NAR, NIR; *narG*, *nirK*), and nitrogen fixation (*nifH*). The specific methods for assaying these enzymes and genes are detailed below.

Protease activity was determined according to the Chinese agricultural standard NY/T 4549. Briefly, crude enzymes were extracted from fresh compost samples using borate buffer (pH 10.5) at 4 °C with shaking (200 rpm, 60 min). After centrifugation (10,000× *g*, 10 min), the supernatant was incubated with 1% (*w*/*v*) casein dissolved in borate buffer (pH 10.5) at 40 °C for 10 min. The reaction was terminated with 0.4 M trichloroacetic acid. The released tyrosine in the filtrate was quantified colorimetrically at 680 nm following the addition of 0.4 M Na_2_CO_3_ and Folin–Ciocalteu reagent. One unit (U) of protease activity was defined as the amount of enzyme producing 1 μg of tyrosine per minute under the assay conditions, expressed per gram of dry weight (μmol Tyr h^−1^ g^−1^) based on the moisture content of the sample.

The activities of AMO, NAR, and NIR were determined using commercial assay kits (AMO, Cat. No. SMHC9; Molfarming, Shanghai, China, NAR/NIR, Cat. Nos. BC3100 and BC2995; Solarbio, Beijing, China). Briefly, AMO activity was assayed by incubating 1.0 g of prepared sample with ammonium sulfate and potassium chlorate at 25 °C for 24 h; activity was quantified based on the enzymatic consumption of ammonium, determined via the indophenol blue method at 630 nm. NAR activity was determined by incubating 0.1 g of sample with nitrate and NADH at 37 °C for 24 h, measuring the enzymatic reduction in NO_3_^−^ to NO_2_^−^. NIR activity was assessed by incubating 0.05 g of sample with nitrite at 25 °C for 3 h, monitoring the enzymatic consumption of NO_2_^−^. For NAR and NIR, NO_2_^−^ concentrations were colorimetrically determined using the Griess reagent at 520 nm or 540 nm, respectively. One U of enzyme activity was defined as the amount of enzyme catalyzing the transformation (consumption of ammonium for AMO; production of NO_2_^−^ for NAR; consumption of NO_2_^−^ for NIR) of 1 μmol of substrate/product per day per gram of sample.

Total genomic DNA was extracted from compost samples using the E.Z.N.A.^®^ Soil DNA Kit (Omega Bio-tek, Norcross, GA, USA) and diluted 10-fold prior to analysis to minimize PCR inhibition by humic substances. Quantitative PCR (qPCR) was performed in triplicate using SYBR^®^ Premix Ex Taq™ II (Takara Bio, Kusatsu, Japan) in 25-μL reactions containing 1 μL of DNA template and gene-specific primers (Table 2) [19,20]. Amplification conditions comprised initial denaturation at 95 °C for 3 min, followed by 40 cycles of 95 °C (30 s), annealing (30 s), and 72 °C (30 s), concluding with a melt curve analysis to confirm amplification specificity. Absolute gene abundances were calculated using standard curves generated from serial dilutions of plasmids (10^2^–10^8^ copies). All assays met quality control standards, exhibiting amplification efficiencies (E) of 90–110% and linearity (*R*^2^) > 0.99 (Supplementary Table S1). Final gene abundances were normalized to total DNA and expressed as (log_10_ copies ng^−1^ DNA) to mitigate biases arising from variable extraction yields during the composting process. Samples with non-detectable amplification were considered as LOD/2 (limit of detection) for the calculation of gene abundances.

### 2.5. Bacterial Community Structure Analysis via 16S rRNA Gene Amplicon Sequencing

Total genomic DNA was extracted from compost samples collected on days 0, 3, 22, 30, and 50 using the FastDNA^®^ SPIN Kit for Soil (MP Biomedicals, Santa Ana, CA, USA). This extraction method was selected to ensure efficient lysis of the thermophilic microflora for community profiling, distinct from the protocol used for functional gene quantification. The V3-V4 hypervariable region of the bacterial 16S rRNA gene was amplified using primers 341F/806R and sequenced on an Illumina NovaSeq 6000 platform (2 × 250 bp) by Gene Denovo Biotechnology Co., Ltd. (Guangzhou, China). Notably, while the commercial decomposer (COM) contained a fungal component (*Aspergillus oryzae*), this study focused specifically on bacterial community dynamics as tracked by the 16S rRNA gene workflow.

Raw sequencing reads were quality-filtered using FASTP v0.18.0 [21] to remove low-quality reads (Q-score < 20) and adapters. Paired-end reads were merged into assembled sequences using FLASH2 v1.2.11 [22]. Denoising and generation of amplicon sequence variants (ASVs) were performed using the UNOISE3 algorithm implemented in Usearch v11.0.667 [23] with default parameters (alpha = 2.0, minsize = 8). Taxonomic assignment of representative ASV sequences was conducted using the RDP Classifier v2.14 [24] against the Silva v138.2 SSU Ref NR 99 database [25] (non-redundant version clustered at 99% similarity) with a minimum confidence threshold of 80% for all taxonomic levels. The relative abundance profiles of the microbial community at the phylum and genus levels were visualized using the ggplot2 package v3.3.5 in R.

To account for uneven sequencing effort, the ASV table was rarefied to a depth of 37,140 sequences per sample (corresponding to the minimum library size) prior to diversity analysis. The dataset (*n* = 45) yielded a mean sequencing depth of 77,682 ± 14,235 effective sequences per sample. Bioinformatic analysis was performed using the OmicSmart platform (Gene Denovo, Guangzhou, China). Specifically, alpha diversity indices (Chao1 richness estimator and Shannon diversity index) were calculated using the vegan package in R (v4.0.3) implemented within the platform’s pipeline based on this rarefied ASV table. Statistical significance was evaluated using One-Way Analysis of Variance (ANOVA) followed by Tukey’s Honestly Significant Difference (HSD) post hoc test (*p* < 0.05, *n* = 3). Comparisons were conducted in two dimensions: (1) to assess differences between treatments at each sampling time point, and (2) to evaluate temporal changes across time points within each treatment. For beta diversity, Unweighted UniFrac distance matrices were computed and visualized using the GUniFrac v1.1 and ape v5.5 packages embedded in the OmicSmart workflow, serving as the basis for Principal Co-ordinates Analysis (PCoA) and UPGMA clustering.

### 2.6. Whole-Genome Sequencing and Annotation of A. pallidus Strain 60

To elucidate the inoculant’s genetic capacity, the complete genome of *A. pallidus* strain 60 was sequenced using a hybrid approach combining long-read (PacBio) and short-read (Illumina) technologies. Genomic DNA was extracted from a pure culture using the HiPure Bacterial DNA Kit (Magen, Guangzhou, China). Long-read SMRTbell libraries (>10 kb) were sequenced on a Pacific Biosciences Sequel system. Short-read libraries (~350 bp insert size) were sequenced on an Illumina NovaSeq 6000 platform (2 × 150 bp paired-end). The de novo assembly of PacBio reads was performed using Falcon v0.3.0 [26] and polished using quality-filtered Illumina reads with Pilon v1.23 [27]. The final genome was annotated using the NCBI Prokaryotic Genome Annotation Pipeline (PGAP) [28], which performs comprehensive identification of protein-coding genes, rRNAs, tRNAs, and other genomic features.

Functional annotation was enriched by aligning protein-coding genes against the NCBI Nr, UniProt/Swiss-Prot, KEGG, GO, and COG databases. To specifically investigate the strain’s potential for protein degradation and nitrogen conservation, a targeted manual curation was performed. For proteolytic enzymes, candidate genes were identified by keyword filtering (search terms: “protease,” “peptidase,” “proteinase,” and “peptide hydrolase”) against the Nr and COG annotation results. For nitrogen metabolism, genes were identified by mapping the genome sequences to the KEGG “Nitrogen metabolism” pathway (ko00910). The identity of key genes and gene families was further verified by cross-referencing Nr and Swiss-Prot annotations. A circular genome map was generated using the CGView Server through the Proksee platform (https://proksee.ca/; accessed on 10 August 2024).

### 2.7. Statistical and Ecological Analyses

Data are presented as mean ± standard error of the mean (SEM) from three biological replicates (*n* = 3). To account for the temporal dynamics of the composting process, statistical differences were assessed by a Two-Way Analysis of Variance (ANOVA) with treatment and sampling time as factors, except for microbial diversity indices (analyzed as described in Section 2.5). Since significant Treatment × Time interactions were detected (*p* < 0.05), pairwise comparisons were conducted using Tukey’s HSD post hoc test (*p* < 0.05). All statistical analyses were conducted using SPSS Statistics v25.0 (IBM SPSS Inc., Chicago, IL, USA). All graphs were generated using GraphPad Prism v10 (GraphPad Software, La Jolla, CA, USA). Handling of non-detects in qPCR data followed the LOD/2 substitution method [29], which was validated by a sensitivity analysis comparing with LOD substitution. While ANOVA on log-transformed data was the primary statistical method, results for genes with frequent non-detects were cross-verified using the non-parametric Kruskal–Wallis’s test to ensure the robustness of the significance findings.

Multivariate ecological analyses were performed to link microbial community structure with environmental variables. Prior to analysis, the species abundance data were Hellinger-transformed to minimize the “arch effect” and reduce the weight of rare taxa. Environmental variables were standardized (Z-score normalization) to eliminate unit disparities. Redundancy analysis (RDA) was conducted using the vegan package v2.5-7 in R to investigate the relationships between dominant genera and the measured physicochemical and functional parameters. The significance of the constraints was tested using a permutation test (999 permutations).

Associations between dominant genera and key functional parameters were explored using Spearman rank correlation. To control for multiple comparisons, raw *p*-values were adjusted using the Benjamini–Hochberg false discovery rate (FDR) procedure, and correlations with an adjusted *p*-value (denoted as *q*) < 0.05 were considered significant. For the co-occurrence network, edge weights were defined by the Spearman correlation coefficient (ρ). To evaluate the robustness of potential edges against compositional bias, a stability analysis was performed using 1000 bootstrap resamples. In each iteration, correlations were recalculated, and edge stability was defined as the frequency with which the correlation remained significant (|ρ| > 0.4, *q* < 0.05). Only edges with a stability index ≥ 80% were retained in the final co-occurrence network, which was visualized using the igraph package v1.2.6 in R.

## 3. Results

### 3.1. Genomic Potential of A. pallidus Strain 60 for Nitrogen Conservation

**Figure 2:**
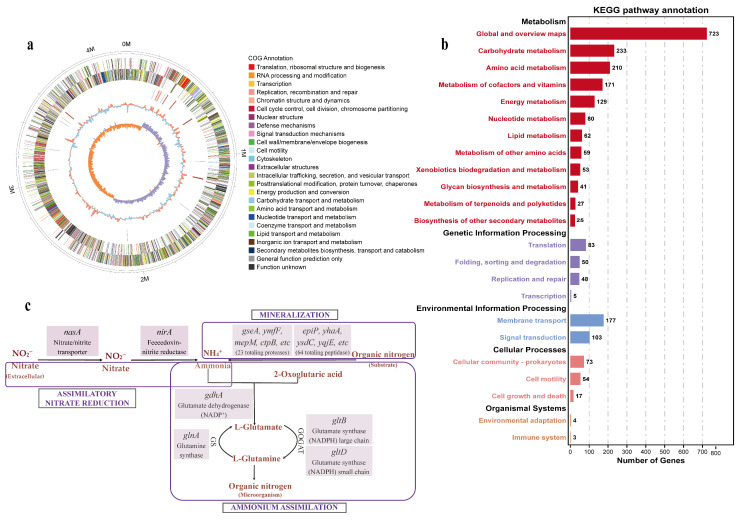
Genomic architecture and nitrogen-metabolic potential of *A. pallidus* strain 60. (**a**) Circular genome map highlighting (from outer to inner rings): coordinate scale, coding sequences (colored by COG categories), non-coding RNAs, GC content, and GC skew. (**b**) Functional classification of protein-coding genes based on KEGG pathways. (**c**) Strain-specific metabolic reconstruction illustrating the specific genetic repertoire for nitrogen conservation and assimilation.

To elucidate the genetic underpinnings of the potential of *A. pallidus* strain 60 as a composting inoculant, the complete genome was sequenced and analyzed. The genome was assembled into a single circular chromosome with no extrachromosomal plasmids detected. The raw sequencing reads (PacBio and Illumina) have been deposited in the NCBI Sequence Read Archive (SRA) under BioProject PRJNA1141263. The complete genome sequence assembly has been assigned GenBank accession number CP166244 (scheduled for public release). The 4.21 Mbp genome contains 3832 predicted protein-coding genes, with a notable specialization in amino acid transport and metabolism (Figure 2a,b; Supplementary Table S2).

A genomic mining approach revealed a comprehensive genetic blueprint for nitrogen conservation in *A. pallidus* strain 60. The strain possesses an extensive genetic toolkit for protein decomposition, including 23 putative proteases and 64 peptidases (Figure 2c, Supplementary Table S3). Notably, the presence of multiple genes encoding secreted enzymes—such as those from the S8 family of serine peptidases, which includes collagen-degrading enzymes in some thermophilic bacteria [30]—provides a direct genetic basis for its potent proteolytic activity in protein-rich substrates.

Beyond protein degradation, the genome is equipped for efficient nitrogen assimilation. We identified a ferredoxin-nitrite reductase gene (*nirA*), supported by nitrate/nitrite transporters, which constitutes the key component of the assimilatory nitrate reduction pathway. This pathway is complemented by the complete suite of genes for GS/GOGAT/GDH pathways, enabling rapid intracellular ammonium assimilation and channeling of nitrogen into biomass. In contrast to these robust nitrogen-assimilating and conserving pathways, genome mining of *A. pallidus* strain 60 alone revealed the absence of key genes for nitrification (*amoA*, *nxrA*) or the gas-forming steps of denitrification (*nirS*/*nirK*, *norB*, *nosZ*). Collectively, these genomic features portray *A. pallidus* strain 60 as a microbial species adept at protein decomposition and nitrogen retention, rather than nitrogen loss.

### 3.2. Physicochemical Parameters and Maturity Evolution

#### 3.2.1. Process Environmental Conditions

**Figure 3:**
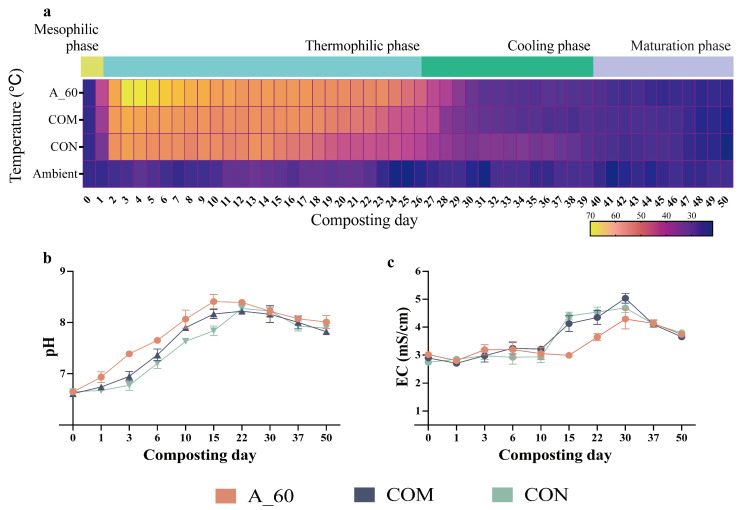
Temporal evolution of environmental conditions during composting. (**a**) Temperature dynamics, with horizontal bars denoting the distinct thermal phases (Mesophilic to Maturation) in the inoculated treatment. (**b**) pH and (**c**) electrical conductivity (EC) profiles. A_60: *A. pallidus* inoculation; COM: commercial decomposer; CON: control. These treatment designations apply to all subsequent figures. Data represent mean ± SEM (*n* = 3). Statistical comparisons are detailed in Supplementary Table S4.

Based on its promising genomic potential, we tested the efficacy of *A. pallidus* strain 60 in a laboratory-scale composting trial. The process, in line with the widely accepted composting model [31], progressed through four distinct phases defined by temperature: mesophilic, thermophilic (>45 °C), cooling (<45 °C), and a maturation phase where temperatures stabilized near ambient levels (Figure 3a). Inoculation with *A. pallidus* (A_60) induced a more intense and prolonged thermophilic phase, achieving a peak temperature of 70 °C, significantly higher than the commercial inoculant (COM, 60 °C) and control (CON, 58 °C) treatments (*p* < 0.05) (Figure 3a). Crucially, the A_60 treatment maintained temperatures ≥ 60 °C for 7 days, satisfying the thermal requirements for pathogen inactivation [32].

Regarding other physicochemical properties, the A_60 and COM treatments reached their peak on day 15, which was earlier than the CON treatment (day 22; Figure 3b). All the treatments yielded the final EC value below the 4.0 mS/cm maturity threshold, meeting the maturity benchmark for salinity safety [33] (Figure 3c).

#### 3.2.2. Nutrient Transformation and Humification

**Figure 4:**
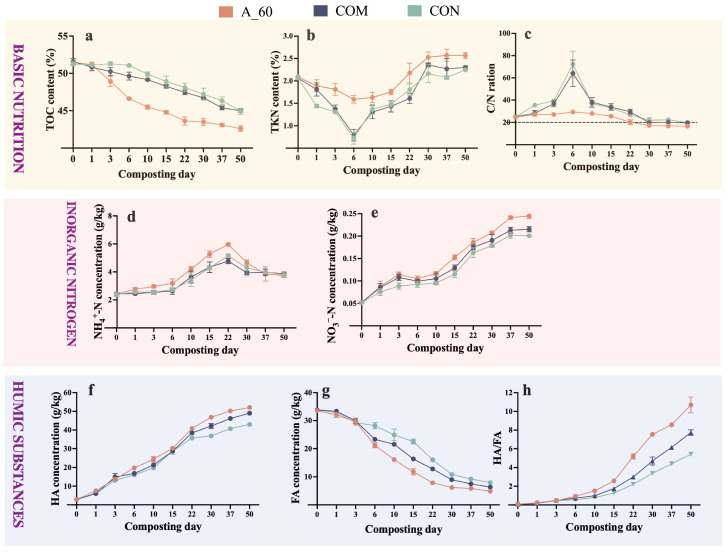
Dynamics of organic matter degradation and nitrogen transformation during composting. The panels display: (**a**–**c**) Basic nutrition (TOC, TKN, C/N ratio), (**d**,**e**) Inorganic Nitrogen (NH_4_^+^-N, NO_3_^−^-N); and (**f**–**h**) Humic Substances: (HA, FA, HA/FA). Data points represent mean ± SEM (*n* = 3). Statistical differences between treatments are detailed in Supplementary Table S4. Shaded background blocks serve as visual separators to distinguish the different nutrient categories.

*A. pallidus* inoculation streamlined the composting process and yielded a superior final product. The A_60 treatment accelerated organic matter mineralization. By the end of composting (day 50), cumulative TOC degradation rates calculated from initial and final contents reached 17.49% (A_60), significantly surpassing the 12.75% and 13.08% rates observed in COM and CON, respectively (*p* < 0.05; Figure 4a). Concurrently, A_60 maintained higher nitrogen levels. At termination (day 50), *A. pallidus* inoculation significantly increased final TKN by 10.87% and 13.33% compared to COM and CON, respectively (*p* < 0.05; Figure 4b). Given identical initial feedstocks, this significant enrichment of TKN serves as an indicator of enhanced nitrogen conservation relative to the controls. Consequently, the C/N ratio in A_60 met the maturity threshold (<20) by day 30, which was 7 and 20 days ahead of COM and CON treatments, respectively (Figure 4c).

Furthermore, the A_60 treatment showed a more intense ammonification phase, with peak NH_4_^+^-N values exceeding COM by 19.83% and CON by 13.28% (*p* < 0.01; Figure 4d). This ammonium was subsequently more effectively converted to stable nitrate (NO_3_^−^-N), with final contents (day 50) exceeding COM and CON by 18.65% and 13.75%, respectively (*p* < 0.01; Figure 4e). *A. pallidus* inoculation also profoundly enhanced humification, leading to significantly higher HA (52.16 g kg^−1^) and lower FA (6.07 g kg^−1^) contents by day 50 (*p* < 0.01; Figure 4f,g). As a result, the humification index (HA/FA) in A_60 exceeded the maturity benchmark of 1.0 by day 10 and culminated at 10.74, markedly surpassing the controls (*p* < 0.01; Figure 4h). These findings demonstrate that *A. pallidus* inoculation not only shortens the composting cycle but also produces a more stable and mature compost.

### 3.3. Microbial Community Dynamics and Succession

**Figure 5:**
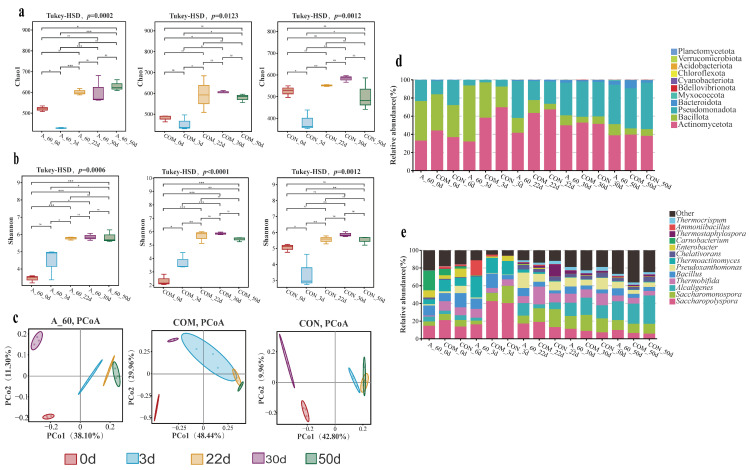
Bacterial community succession and diversity shifts. (**a**,**b**) Temporal dynamics of Alpha diversity indices (Chao1 and Shannon). Asterisks indicate significant differences between treatments at specific time points (* *p* < 0.05, ** *p* < 0.01; *** *p* < 0.01, ns *p* > 0.05). Data are presented as mean ± SEM (*n* = 3). (**c**) Beta diversity visualization via Principal Co-ordinates Analysis (PCoA) based on Unweighted UniFrac distances. (**d**,**e**) Taxonomic composition profiles at the (**d**) phylum and (**e**) genus levels. The UPGMA clustering tree and detailed cross-treatment statistical comparisons of alpha diversity (One-Way ANOVA with Tukey’s HSD) are provided in Supplementary Figure S2.

To understand the microbial basis for these process improvements, we analyzed the bacterial community dynamics using 16S rRNA gene amplicon sequencing. The raw sequencing data have been deposited in the NCBI SRA under BioProject PRJNA1426577 (scheduled for public release upon publication). While the alpha diversity (Chao1 and Shannon indices) did not consistently differ between the A_60 treatment and the controls across the entire process (except for specific variations on days 0 and 50, see cross-treatment comparisons in Supplementary Figure S2), the temporal dynamics of these diversity indices were profoundly altered by the composting stages (Figure 5a,b). Principal Co-ordinates Analysis (PCoA) (Figure 5c) and UPGMA clustering (Supplementary Figure S2) both showed that while composting time was the primary driver of community shifts, especially those from the early thermophilic stages formed distinct clusters.

This divergence was driven by a two-stage microbial community succession. During the early thermophilic phase (day 3), the A_60 treatment fostered a community dominated by Bacillota (61.75% relative abundance) (Figure 5d), driven by the significant enrichment of two genera known for proteolytic activity: *Thermoactinomyces* (23.51% in A_60 vs. <17.1% in controls; *p* < 0.05) and *Ammoniibacillus* (16.83% vs. ≤2.0% in controls; *p* < 0.01) (Figure 5e). Conversely, the controls were dominated by Actinomycetota in COM (58.38%) and CON (69.85%) groups (Figure 5d), largely due to a higher abundance of *Saccharopolyspora* (Figure 5e). As the compost matured, a key functional distinction emerged: by day 22, the genus *Pseudoxanthomonas,* which has been associated with nitrogen transformation in previous studies [34], became dominant in the A_60 treatment, succeeding the early-stage decomposers, while *Saccharopolyspora* persisted in the controls (Figure 5e).

### 3.4. Functional Gene Dynamics of Nitrogen Transformation

**Figure 6:**
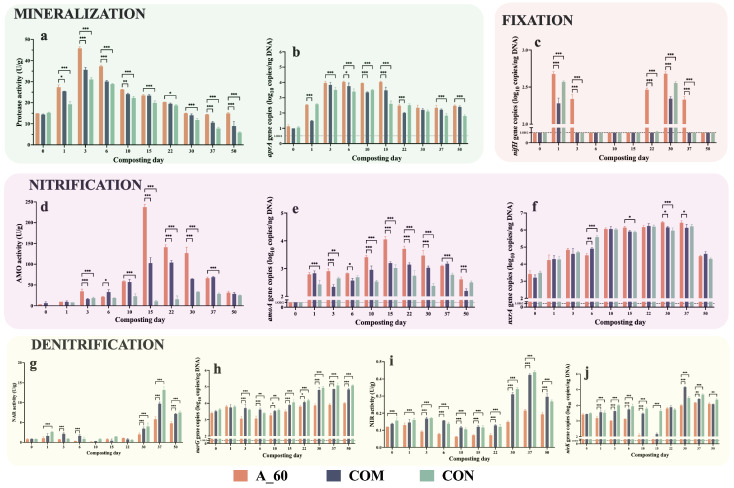
Dynamics of enzymatic activity and gene abundance across nitrogen cycling pathways. Panels are grouped by functional step: (**a**,**b**) Organic nitrogen mineralization (Protease activity and *aprA* gene); (**c**) Nitrogen fixation (*nifH* gene); (**d**–**f**) Nitrification (AMO activity, *amoA* and *nxrA* genes); and (**g**–**j**) Denitrification (NAR/NIR activities, *narG* and *nirK* genes); (**j**). Asterisks indicate significant differences between treatments at specific time points (* *p* < 0.05, ** *p* < 0.01, *** *p* < 0.001; Mean ± SEM, *n* = 3). Non-detects were treated as LOD/2 for statistical analysis (a sensitivity analysis was performed to compare with LOD substitution). Bars at the baseline level indicate groups with no detectable signal across replicates. Shaded background blocks distinguish the different functional pathways.

Consistent with the two-stage microbial succession described above, the functional dynamics of nitrogen transformation also exhibited a clear temporal shift (Figure 6). The process was initiated by a surge in organic nitrogen mineralization, with protease activity peaking across all treatments in the early thermophilic phase (day 3). However, aligning with the enrichment of proteolytic genera, the A_60 treatment exhibited a significantly more potent proteolytic response, evidenced by both a higher peak activity and a more sustained, elevated abundance of the *aprA* gene (*p* < 0.01) (Figure 6a,b).

Following the community shift, the functional profile transitioned towards nitrogen transformation. The activity of AMO and the abundance of its encoding gene, *amoA*, peaked in the late thermophilic phase and the subsequent cooling phase (days 15–37). Throughout this period, the A_60 treatment consistently maintained significantly higher functional activity and genetic potential for this key nitrification step (*p* < 0.01) (Figure 6c,d). The genetic capacity for the subsequent step, nitrite oxidation, based on *nxrA* gene abundance, was also significantly higher at key time points in the A_60 treatment (Figure 6e).

In contrast, pathways for nitrogen loss were actively suppressed in the A_60 treatment. While a general trend of increasing denitrification potential was observed across all treatments during the cooling and maturation phases (days 30–50), the A_60 treatment showed significantly lower activities of NAR and NIR, and lower abundances of their encoding genes (*narG*, *nirK*), for most of the process (*p* < 0.01) (Figure 6f–i). In addition, the nitrogen fixation gene (*nifH*) exhibited a higher frequency of detection in the A_60 treatment (detectable at five distinct sampling points: days 1, 3, 22, 30, and 37) compared to the sporadic signals observed in the COM and CON groups (Figure 6j). While the high-nitrogen environment likely inhibits active fixation [35], the persistence of this gene suggests that the microbial consortium shaped by *A. pallidus* inoculation maintained a distinct genetic reservoir for nitrogen cycling compared to the controls.

### 3.5. Multivariate Integration of Community and Function

**Figure 7:**
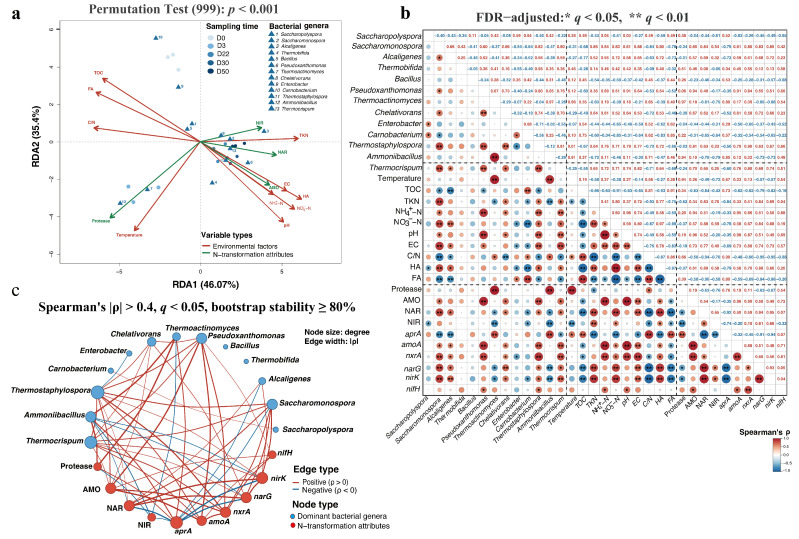
Multivariate associations linking microbial community structure to nitrogen transformation functions in the A_60 treatment. (**a**) RDA biplot correlating community succession (samples/genera) with environmental variables. (**b**) Spearman correlation heatmap identifying specific linkages between dominant genera and functional attributes (* *q* (FDR-adjusted *p*-value) < 0.05, ** *q* < 0.01). (**c**) Co-occurrence network visualizing the statistically robust interactions (|ρ| > 0.4, *q* < 0.05; stability index ≥ 80%) between taxa (blue nodes) and nitrogen functions (red nodes).

To elucidate the specific mechanistic associations between bacterial community structure and nitrogen transformation functions within the inoculated system, multivariate analyses were performed on the A_60 treatment (Figure 7). RDA confirmed that the microbial community in this treatment was tightly coupled to the composting process variables, with the first two axes explaining 81.47% of the total variation (*p* < 0.001) (Figure 7a). The biplot revealed a clear temporal trajectory: samples from the early thermophilic phase (day 3), where bacterial community divergence was greatest, were strongly associated with temperature and protease activity, aligning with the position of the key early-stage genera, *Thermoactinomyces* and *Ammoniibacillus*. Later-stage samples correlated strongly with nitrification functions (AMO) and NO_3_^−^-N content, associating with the late-stage dominant genus, *Pseudoxanthomonas*.

Spearman correlation analysis (based on *n* = 15 matched samples with FDR adjustment) within the A_60 treatment revealed strong statistical associations between microbial taxa and nitrogen transformation functions (Figure 7b). Both *Thermoactinomyces* and *Ammoniibacillus* exhibited significant positive correlations with protease activity (ρ = 0.91 and 0.94, respectively; *q* < 0.05) and the *aprA* gene (ρ = 0.80 and 0.85; *q* < 0.05). In the later stages, *Pseudoxanthomonas* showed consistent associations with nitrification markers, exhibiting strong positive correlations with AMO activity (ρ = 0.94), *amoA* abundance (ρ = 0.90) and *nxrA* abundance (ρ = 0.84) (all *q* < 0.05).

A co-occurrence network was constructed to visualize these interactions. To ensure robustness given the sample size, edges were retained only if they satisfied both FDR significance (*q* < 0.05) and high bootstrap stability (≥80% over 1000 iterations). The resulting network, comprising 21 nodes and 59 edges (Figure 7c), synthesized the orchestrated microbial succession from early-stage decomposers to a late-stage hub genus linked to nitrogen conservation (Figure 7c). This statistically supported link highlights an orchestrated succession where specific taxa are strongly correlated with nitrogen transformation rates, providing a statistical basis for the metabolic dynamics observed in the *A. pallidus*-inoculated process.

## 4. Discussion

This study demonstrates that bioaugmentation with *A. pallidus* strain 60, an in-house isolate, may represent an effective strategy for accelerating maturation and enhancing relative nitrogen conservation in laboratory-scale MBM composting. Genomic analysis revealed a metabolic capability for organic matter decomposition and nitrogen cycling. This potential was corroborated by the composting trial, where the inoculant initiated a rapid thermal surge and proteolytic cascade—traits predicted by its genome and confirmed by physicochemical monitoring. By modifying the composting niche through these activities, *A. pallidus* facilitated an orchestrated, two-stage microbial succession. These findings support the hypothesis that *A. pallidus* functions as a microbial driver in this system.

Our investigation was motivated by the genomic portrait of *A. pallidus* strain 60 as a promising nitrogen-retaining agent. Its rich inventory of genes for extracellular proteases provided a strong rationale for its application in a protein-dense composting substrate. Furthermore, the genomic evidence indicates a strategy for nitrogen conservation centered on efficient assimilation. The identified genetic toolkit—including the assimilatory nitrate reduction pathway (centered on the *nirA* gene) and the concurrent GS/GOGAT/GDH systems for rapid ammonium immobilization—enables the strain to efficiently channel nitrogen liberated from proteolysis into cellular biomass, a process supported by established roles of these pathways [36,37,38]. We therefore posited that these coordinated mechanisms would minimize the release of inorganic nitrogen intermediates, thereby reducing the potential for nitrogen loss. This powerful genetic potential led us to hypothesize that *A. pallidus* strain 60 could not only initiate decomposition but also positively modulate the nitrogen economy of the composting system.

The composting trial provided observations consistent with this hypothesis. The primary role of *A. pallidus* appears to be environmental modification. Its initial metabolic burst, enabled by its proteolytic genes and confirmed by the surge in protease activity, generated a powerful thermogenic response (Figure 3 and Figure 6). This activity established a selective niche characterized by high temperature and ammonium availability—environmental filters known to shape microbial consortia in composting [39].

This process exemplifies the ecological concept of niche construction, where organisms actively alter their local habitat, thereby influencing the community structure and function [40]. Rather than sustaining dominance, the inoculant primarily served as a catalyst, initiating environmental changes that facilitated the establishment of a specialized microbial assembly through ecological selection.

This environmental modification was followed by the observed two-stage microbial succession. The first stage was dominated by proteolytic thermophiles, *Thermoactinomyces* and *Ammoniibacillus*. Our analysis identified these genera as the dominant taxa temporally associated with the proteolytic phase (Figure 5 and Figure 7). Their potential role is substantiated by previous reports on potent protease secretion and high ammonia tolerance [41,42,43,44]. We suggest that their rapid enrichment was likely a direct consequence of the strong thermal selection pressure exerted by the inoculant-driven temperature surge (70 °C), which created a favorable niche for these extreme thermophiles. Consequently, the rapid assembly of this group is consistent with the accelerated ammonification observed in the A_60 treatment (Figure 4).

Subsequently, a distinct shift occurred to a second-stage community led by *Pseudoxanthomonas*. Our data showed a strong correlation between this genus and nitrification markers (Figure 6 and Figure 7), consistent with previous studies associating *Pseudoxanthomonas* with nitrogen retention in other ecosystems [34]. Given that our genomic analysis confirmed *A. pallidus* lacks key nitrification genes (*amoA*, *nxrA*), the elevated nitrification activity observed in the A_60 treatment indicates that the inoculant did not perform this function directly. Instead, it facilitated the enrichment of native nitrifiers. This structured succession from primary decomposers to putative nitrogen managers represents the likely ecological process contributing to the enhanced performance of the bioaugmented system. Thus, rather than acting in isolation, *A. pallidus* likely functioned as an environmental modifier, establishing conditions that favored the subsequent development of a nitrogen-conserving microbiome.

Placing these findings in the context of broader microbiome engineering, our results align with recent studies targeting nitrogen conservation in animal-derived waste composting. For instance, a recent study demonstrated that adding exogenous microbial agents to pig carcass compost significantly prolonged the high-temperature period and regulated nitrogen functional genes (e.g., *amoA*, *glnA*), ultimately increasing total nitrogen and nitrate content [45]. Our findings mirror this pattern. However, regarding the biological drivers, the role of *Sphingobacterium* and *Paenalcaligenes* was highlighted in that study, whereas our study identified a succession dominated by *Thermoactinomyces*, *Ammoniibacillus*, and *Pseudoxanthomonas*. This comparison suggests that while the functional outcome (nitrogen conservation via prolonged thermophilic conditions) is consistent across bioaugmented carcass composting, the specific taxonomic pathways can be distinct and are likely shaped by the specific inoculant used.

Finally, the performance advantage of *A. pallidus* over the commercial comparator (COM) warrants careful interpretation regarding inoculant composition. Unlike the single-strain bacterial inoculant, the COM consortium included a fungal component (*Aspergillus oryzae*). It is plausible that the rapid thermal surge (>60 °C) initiated by the proteolytic activity in the early phase may have inhibited the mesophilic fungal component of the commercial blend, potentially limiting its contribution to the process [46]. Consequently, our findings highlight the efficacy of *A. pallidus* specifically within high-temperature composting niches, rather than implying a categorical superiority over bacterial–fungal consortia. Future studies employing multi-kingdom profiling (16S and ITS) are essential to rigorously evaluate the potential synergies between thermophilic bacteria and fungi in engineered mixtures.

## 5. Conclusions

This study demonstrates that bioaugmentation with *A. pallidus* strain 60 significantly accelerated the maturation of laboratory-scale MBM compost and resulted in higher final relative nitrogen and humic acid contents compared to non-inoculated controls and the specific commercial reference tested. Physicochemical and microbial profiling indicate that these improvements were likely driven not by the sustained dominance of the inoculant itself, but by its role in initiating environmental changes—specifically a rapid thermal and proteolytic surge—that facilitated a distinct two-stage microbial succession. The community trajectory shifted from early-stage proteolytic taxa (*Thermoactinomyces*) to late-stage clusters associated with nitrification (*Pseudoxanthomonas*). Based on these findings, we recommend that practitioners prioritize early-stage inoculation (at 1.0 × 10^8^ CFU kg^−1^ dry weight) for the valorization of protein-dense organic wastes to maximize the initial thermogenic proteolytic phase, which establishes the necessary foundation for subsequent relative nitrogen retention. While the observed gene abundances and enzyme activities are consistent with enhanced nitrogen retention, future research should prioritize metatranscriptomic profiling, particularly at critical transition points (e.g., days 3 and 22), to verify whether specific taxa like *Thermoactinomyces* and *Pseudoxanthomonas* actively upregulate functional genes in situ.

## Figures and Tables

**Table 1:** Physicochemical properties of the feedstock for the composting study.

Feedstock	Moisture Content (%, dw) *	Total Organic Carbon (%, dw)	Total Kjeldahl Nitrogen (%, dw)	C/N Ratio
MBM	12.50 ± 0.84	52.51 ± 0.27	7.80 ± 0.19	6.73 ± 0.21
wheat straw	9.00 ± 0.31	51.18 ± 0.32	0.65 ± 0.07	78.74 ± 7.85

* dw denotes dry weight.

**Table 2:** Primer sets for nitrogen transformation functional genes in qPCR analysis.

Gens	Primers	Primer Sequences * (5′—3′)	Size (bp)	Annealing Temperature (°C)
*aprA*	aprA-F1	CGACACATTGCCCTTCAACC	193	55 °C
	aprA-R1	GGTGTACGGCTTCAACTCCA		
*amoA*	amoA-1F amoA-1R	GGGGTTTCTACTGGTGGT CCCCTCKGSAAAGCCTTCTTC	491	58 °C
*nxrA*	nxrA-1F nxrA-1R	CAGACCGACGTGTGCGAAAG TCYACAAGGAACGGAAGGTC	322	52 °C
*nirK*	nirK-F nirK-R	TCATGGTGCTGCCGCGYGANGG GAACTTGCCGGTKGCCCAGAC	326	55 °C
*narG*	narG-F narG-R	TAGTGGGCAGGAAAAC CGTAGAAGAAGCTGGTGCTGTT	110	52 °C
*nifH*	Po1F Po1R	TGCGAYCCSAARGCBGACTC ATSGCCATCATYTCRCCGGA	360	58 °C

* S represents C or G; Y represents C or T; K represents G or T; R represents A or G; B represents C, G or T; N represents A, C, T or G.

## Data Availability

The raw 16S rRNA gene amplicon sequencing data presented in this study are available in the NCBI Sequence Read Archive (SRA) under BioProject accession number PRJNA1426577. The raw genomic sequencing reads (PacBio and Illumina) for *Aeribacillus pallidus* strain 60 are publicly available under BioProject PRJNA1141263. The complete genome sequence assembly has been deposited in GenBank under accession number CP166244 (scheduled for public release). The complete physicochemical metadata and raw qPCR cycle threshold (Ct) values are provided in Supplementary Table S5.

## Supplementary Materials

The following supporting information can be downloaded at https://www.mdpi.com/article/10.3390/microorganisms14030589/s1, Supplementary Table S1. Standard curve parameters (efficiency and linearity) used for quantitative real-time PCR (qPCR) assays; Supplementary Table S2. Genomic features and functional classification of predicted protein-coding genes in Aeribacillus pallidus strain 60; Supplementary Table S3. Inventory of putative proteolytic and nitrogen transformation-related functional proteins identified in the genome of Aeribacillus pallidus strain 60; Supplementary Table S4. Detailed statistical analysis results for physicochemical parameters and maturity indices; Supplementary Table S5. Source data for physicochemical properties, enzymatic activities, and functional gene abundances; Supplementary Figure S1. Schematic of experimental unit dimensions and spatial sampling strategy; Supplementary Figure S2. Hierarchical clustering and detailed statistical comparisons of alpha diversity.
