# Supplementary material for: Aeribacillus pallidus Inoculant Orchestrates Functional Microbial Succession for Enhanced Nitrogen Transformation in High-Protein Waste Composting
Source: Microorganisms. 2026 Mar 5;14(3):589. doi: 10.3390/microorganisms14030589 (PMC13029174; doi:10.3390/microorganisms14030589)
Supplement: Supplementary file 1 [file microorganisms-14-00589-s001.zip › Supplementary Table S1-S3 & Figure S1-S2.pdf]

Supplementary Table S1. Standard curve parameters (efficiency and linearity) used for quantitative real-time PCR (qPCR) assays.

Genes	Standard curve	correlation coefficients (R^2)	Slope	Efficiencies (E, %)
aprA	Y= -3.1537 log X+ 37.14	0.9962	-3.15	107.7%
amoA	Y= -3.2521 log X+ 41.664	0.9974	-3.30	100.9%
nxrA	Y= -3.2865 log X+ 43.443	0.9939	-3.28	101.8%
narG	Y= -3.3258 log X+ 46.38	0.9969	-3.32	100.9%
nirK	Y= -3.1142 log X+ 42.254	0.9903	-3.12	109.2%
nifH	Y= -3.093 log X+ 42.203	0.9948	-3.09	110.7%

Supplementary Table S2. Genomic features and functional classification of predicted protein-coding genes in *Aeribacillus pallidus* strain 60.

Genome size/Mbp	4.21
GC content/%	39.44
Genes Predicted	3 832
tRNAs Copies	82
16S rRNAs Copies	7
23S rRNAs Copies	7
5S rRNAs Copies	6
sRNAs Copies	5
Annotated Genes in Nr	3 780
Annotated Genes in Swissport	2 708
Annotated Genes in COG	2 681
Annotated Genes in K EGG	2 255
Total Annotation Genes	3 782

Supplementary Table S3. Inventory of putative proteolytic and nitrogen transformation-related functional proteins identified in the genome of *Aeribacillus pallidus* strain 60.

ID	Nr-ID	Nr-annotation	COG-ID	COG-Function-Description
PEPTIDE/PROTEIN UTILIZATION				
AB6A62_00315	BBU41236.1	minor extracellular protease vpr *[Aeribacillus pallidus]*	COG1404	Subtilisin-like serine proteases
AB6A62_00510	WP_094244734.1	site-2 protease family protein *[Aeribacillus pallidus]*	COG1994	Zn-dependent proteases
AB6A62_01220	REJ16399.1	serine protease *[Aeribacillus pallidus]*	COG0265	Trypsin-like serine proteases, typically periplasmic, contain C-terminal PDZ domain
AB6A62_01310	WP_094244813.1	spore protease YyaC [*Aeribacillus pallidus*]	--	--
AB6A62_01955	AXI38622.1	ATP-dependent Clp protease ATP-binding subunit [*Aeribacillus pallidus*]	COG0542	ATPases with chaperone activity, ATP-binding subunit
AB6A62_02760	WP_063386522.1	CPBP family intramembrane metalloprotease *[Aeribacillus pallidus]*	--	--
AB6A62_03175	AXI38746.1	rhomboid family intramembrane serine protease [*Aeribacillus pallidus*]	COG0705	Uncharacterized membrane protein (homolog of Drosophila rhomboid)
AB6A62_03385	WP_066248103.1	CPBP family intramembrane metalloprotease [*Aeribacillus pallidus*]	COG1266	Predicted metal-dependent membrane protease
AB6A62_03555	KZM56027.1	hydrogenase maturation protease [*Aeribacillus pallidus*]	--	--
AB6A62_03560	WP_063387612.1	hydrogenase maturation protease *[Aeribacillus pallidus]*	COG0680	Ni,Fe-hydrogenase maturation factor
AB6A62_06280	WP_064549869.1	Clp protease ClpP [Parageobacillus thermoglucosidasius]	COG0740	Protease subunit of ATP-dependent Clp proteases
AB6A62_06590	WP_144596680.1	trypsin-like serine protease [Aeribacillus composti]	COG3591	V8-like Glu-specific endopeptidase
AB6A62_08805	KZM56211.1	ATP-dependent Clp protease ATP-binding subunit [*Aeribacillus pallidus*]	COG0542	ATPases with chaperone activity, ATP-binding subunit
AB6A62_12650	WP_126410377.1	Clp protease ClpP [Bacillus yapensis]	COG0616	Periplasmic serine proteases (ClpP class)
AB6A62_13070	KZN97657.1	zinc protease [*Aeribacillus pallidus*]	COG0612	Predicted Zn-dependent peptidases
AB6A62_13210	AXI39808.1	RIP metalloprotease RseP *[Aeribacillus pallidus]*	COG0750	Predicted membrane-associated Zn-dependent proteases 1
AB6A62_13420	AXI39850.1	ATP-dependent protease subunit HslV *[Aeribacillus pallidus]*	COG5405	ATP-dependent protease HslVU (ClpYQ), peptidase subunit
AB6A62_14485	WP_094246146.1	glutamic-type intramembrane protease PrsW [*Aeribacillus pallidus*]	COG2339	Predicted membrane protein
AB6A62_14520	KZN95881.1	CAAX protease *[Aeribacillus pallidus]*	COG1266	Predicted metal-dependent membrane protease

AB6A62_15215	REJ25359.1	rhomboid family intramembrane serine protease [*Aeribacillus pallidus*]	COG0705	Uncharacterized membrane protein (homolog of Drosophila rhomboid)
AB6A62_15945	AXI40228.1	ribosomal-processing cysteine protease Prp [*Aeribacillus pallidus*]	COG2868	Predicted ribosomal protein
AB6A62_16090	REJ21337.1	ATP-dependent protease LonB [*Aeribacillus pallidus*]	COG1067	Predicted ATP-dependent protease
AB6A62_16095	AXI40259.1	ATP-dependent protease ATP-binding subunit ClpX [*Aeribacillus pallidus*]	COG1219	ATP-dependent protease Clp, ATPase subunit
AB6A62_00235	WP_117016854.1	M23 family metallopeptidase *[Aeribacillus pallidus]*	COG0739	Membrane proteins related to metalloendopeptidases
AB6A62_00595	REJ22759.1	L,D-transpeptidase [*Aeribacillus pallidus*]	COG1376	Uncharacterized protein conserved in bacteria
AB6A62_01435	WP_117017543.1	D-alanyl-D-alanine carboxypeptidase [*Aeribacillus pallidus*]	COG1686	D-alanyl-D-alanine carboxypeptidase
AB6A62_01600	REJ15229.1	sporulation peptidase YabG [*Aeribacillus pallidus*]	--	--
AB6A62_01740	REJ13079.1	ATP-dependent metallopeptidase FtsH/Yme1/Tma family protein [*Aeribacillus pallidus*]	COG0465	ATP-dependent Zn proteases
AB6A62_01875	WP_066247876.1	A24 family peptidase [*Aeribacillus pallidus*]	COG1989	Type II secretory pathway, prepilin signal peptidase PulO and related peptidases
AB6A62_02210	AXI38673.1	type I methionyl aminopeptidase [*Aeribacillus pallidus*]	COG0024	Methionine aminopeptidase
AB6A62_03445	REJ21880.1	peptidase M48 [*Aeribacillus pallidus*]	--	--
AB6A62_03780	WP_212967285.1	VanY-A/VanY-F/VanY-M family D-Ala-D-Ala carboxypeptidase [Lederbergia ruris]	COG1876	D-alanyl-D-alanine carboxypeptidase
AB6A62_03930	ASS90083.1	signal peptidase I [*Aeribacillus pallidus*]	COG0681	Signal peptidase I
AB6A62_04755	WP_063389226.1	L,D-transpeptidase [*Aeribacillus pallidus*]	COG1376	Uncharacterized protein conserved in bacteria
AB6A62_04920	WP_063389195.1	M23 family metallopeptidase [*Aeribacillus pallidus*]	COG0739	Membrane proteins related to metalloendopeptidases
AB6A62_04925	WP_063389194.1	S41 family peptidase [*Aeribacillus pallidus*]	COG0793	Periplasmic protease
AB6A62_05140	AXI39251.1	ATP-dependent Clp endopeptidase, proteolytic subunit ClpP [*Aeribacillus pallidus*]	COG0740	Protease subunit of ATP-dependent Clp proteases
AB6A62_05625	TVZ82197.1	murein DD-endopeptidase MepM/ murein hydrolase activator NlpD [*Aeribacillus composti*]	COG0739	Membrane proteins related to metalloendopeptidases
AB6A62_05745	WP_130157057.1	leucyl aminopeptidase [*Aeribacillus pallidus*]	COG0260	Leucyl aminopeptidase
AB6A62_05920	WP_063387044.1	zinc metallopeptidase [*Aeribacillus pallidus*]	COG2738	Predicted Zn-dependent protease
AB6A62_06460	KZN97702.1	peptidase [*Aeribacillus pallidus*]	COG1506	Dipeptidyl aminopeptidases/acylaminoacyl-peptidases
AB6A62_06650	WP_144596678.1	dipeptidase PepV [*Aeribacillus composti*]	COG0624	Acetylornithine deacetylase/Succinyl-diaminopimelate desuccinylase and

				related deacylases
AB6A62_06720	WP_063386613.1	M42 family metallopeptidase [*Aeribacillus pallidus*]	COG1363	Cellulase M and related proteins
AB6A62_06780	WP_094245304.1	signal peptidase I [*Aeribacillus pallidus*]	COG0681	Signal peptidase I
AB6A62_07180	WP_063386997.1	oligoendopeptidase F [*Aeribacillus pallidus*]	COG1164	Oligoendopeptidase F
AB6A62_07500	WP_106383560.1	peptidase domain-containing ABC transporter [Bacillus wiedmannii]	COG2274	ABC-type bacteriocin/lantibiotic exporters, contain an N-terminal double-glycine peptidase domain
AB6A62_07660	WP_094245385.1	C39 family peptidase [*Aeribacillus pallidus*]	COG4990	Uncharacterized protein conserved in bacteria
AB6A62_07890	ASS90641.1	peptidase [*Aeribacillus pallidus*]	COG0693	Putative intracellular protease/amidase
AB6A62_07965	WP_232515748.1	ImmA/IrrE family metallo-endopeptidase [*Aeribacillus pallidus*]	--	--
AB6A62_08450	WP_066251054.1	dipeptidase [*Aeribacillus pallidus*]	COG2355	Zn-dependent dipeptidase, microsomal dipeptidase homolog
AB6A62_08700	WP_130156959.1	penicillin-binding transpeptidase domain-containing protein [*Aeribacillus pallidus*]	COG0768	Cell division protein FtsI/penicillin-binding protein 2
AB6A62_09705	REJ16189.1	aminopeptidase [*Aeribacillus pallidus*]	COG2309	Leucyl aminopeptidase (aminopeptidase T)
AB6A62_10080	REJ14890.1	D-alanyl-D-alanine carboxypeptidase family protein [*Aeribacillus pallidus*]	COG1876	D-alanyl-D-alanine carboxypeptidase
AB6A62_10085	WP_232515754.1	S41 family peptidase [*Aeribacillus pallidus*]	COG0793	Periplasmic protease
AB6A62_10115	WP_130157582.1	peptidoglycan endopeptidase [*Aeribacillus pallidus*]	COG0791	Cell wall-associated hydrolases (invasion-associated proteins)
AB6A62_11945	WP_225002292.1	signal peptidase II [*Priestia flexa*]	COG0597	Lipoprotein signal peptidase
AB6A62_12780	WP_144597895.1	ImmA/IrrE family metallo-endopeptidase [Aeribacillus composti]	--	--
AB6A62_13415	WP_094246051.1	HslU--HslV peptidase ATPase subunit [*Aeribacillus pallidus*]	COG1220	ATP-dependent protease HslVU (ClpYQ), ATPase subunit
AB6A62_13475	AXI39861.1	signal peptidase I [*Aeribacillus pallidus*]	COG0681	Signal peptidase I
AB6A62_13750	ASS91631.1	signal peptidase II [*Aeribacillus pallidus*]	COG0597	Lipoprotein signal peptidase
AB6A62_13810	REJ20385.1	sigma-E processing peptidase SpoIIGA [*Aeribacillus pallidus*]	--	--
AB6A62_14055	ASS91688.1	carboxypeptidase M32 [*Aeribacillus pallidus*]	COG2317	Zn-dependent carboxypeptidase
AB6A62_14615	AXI40066.1	D-alanyl-D-alanine carboxypeptidase [*Aeribacillus pallidus*]	COG1686	D-alanyl-D-alanine carboxypeptidase
AB6A62_14710	REJ17544.1	D-alanyl-D-alanine carboxypeptidase [*Aeribacillus pallidus*]	COG1686	D-alanyl-D-alanine carboxypeptidase

AB6A62_14900	WP_063388143.1	MULTISPECIES: tripeptidase T [*Aeribacillus*]	COG2195	Di- and tripeptidases
AB6A62_14950	WP_063388134.1	SpoIVB peptidase [*Aeribacillus pallidus*]	COG0750	Predicted membrane-associated Zn-dependent proteases 1
AB6A62_15065X -Pro	KZM56825.1	Xaa-Pro dipeptidase [*Aeribacillus pallidus*]	COG0006	Xaa-Pro aminopeptidase
AB6A62_15195	WP_063388089.1	M14 family metallocarboxypeptidase [*Aeribacillus pallidus*]	COG2866	Predicted carboxypeptidase
AB6A62_15575	AXI40517.1	GPR endopeptidase [*Aeribacillus pallidus*]	--	--
AB6A62_15690	WP_066250540.1	U32 family peptidase [*Aeribacillus pallidus*]	COG0826	Collagenase and related proteases
AB6A62_15695	WP_063387999.1	U32 family peptidase [*Aeribacillus pallidus*]	COG0826	Collagenase and related proteases
AB6A62_15960	WP_144597693.1	M50 family metallopeptidase [*Aeribacillus composti*]	COG1994	Zn-dependent proteases
AB6A62_15965	WP_063387950.1	M23 family metallopeptidase [*Aeribacillus pallidus* s]	COG0739	Membrane proteins related to metalloendopeptidases
AB6A62_16085	KZM54963.1	endopeptidase La [*Aeribacillus pallidus*]	COG0466	ATP-dependent Lon protease, bacterial type
AB6A62_16300	REJ20711.1	M42 family peptidase [*Aeribacillus pallidus*	COG1363	Cellulase M and related proteins
AB6A62_16595	WP_035065093.1	peptidase M26 [Anoxybacillus gonensis]	COG3210	Large exoproteins involved in heme utilization or adhesion
AB6A62_16665	WP_094246342.1	signal peptide peptidase SppA [*Aeribacillus pallidus*]	COG0616	Periplasmic serine proteases (ClpP class)
AB6A62_17010	WP_063387333.1	aminopeptidase [*Aeribacillus pallidus*]	COG2309	Leucyl aminopeptidase (aminopeptidase T)
AB6A62_17025	REJ23558.1	peptidase M48[*Aeribacillus pallidus*]	COG0501	Zn-dependent protease with chaperone function
AB6A62_18105	WP_094244548.1	trypsin-like peptidase domain-containing protein [*Aeribacillus pallidus*]	COG0265	Trypsin-like serine proteases, typically periplasmic, contain C-terminal PDZ domain
AB6A62_18240	AXI38428.1	peptidase M48 Ste24p [*Aeribacillus pallidus*]	COG0501	Zn-dependent protease with chaperone function
AB6A62_18300	WP_094244528.1	S8 family serine peptidase [*Aeribacillus pallidus*]	COG1404	Subtilisin-like serine proteases
AB6A62_18780	WP_144598489.1	S8 family serine peptidase [*Aeribacillus pallidus*]	COG1404	Subtilisin-like serine proteases
AB6A62_19185	WP_066250069.1	M20 family metallopeptidase [*Aeribacillus pallidus*]	COG1473	Metal-dependent amidase/aminoacylase/carboxypeptidase
AB6A62_19405	WP_120031481.1	membrane dipeptidase [Bacillus sp. PK3_68]	COG2355	Zn-dependent dipeptidase, microsomal dipeptidase homolog
AB6A62_19680	WP_094244400.1	ImmA/IrrE family metallo-endopeptidase [*Aeribacillus pallidus*]	--	--
AB6A62_20705	AKU26558.1	peptidase C14 [Geobacillus sp. LC300]	COG4249	Uncharacterized protein containing caspase domain
NITRITE REDUCTASE				
AB6A62_13675	AXI39901.1	ferredoxin--nitrite reductase [*Aeribacillus pallidus*]	COG0155	Sulfite reductase, beta subunit (hemoprotein)
AB6A62_09335	KZM55344.1	nitrate ABC transporter permease [*Aeribacillus pallidus*]	COG0600	ABC-type nitrate/sulfonate/bicarbonate transport system, permease component
URECYCLE				

AB6A62_09140	WP_063386856.1	urea ABC transporter substrate-binding protein [*Aeribacillus pallidus*]	COG0683	ABC-type branched-chain amino acid transport systems, periplasmic component
AB6A62_09145	WP_094245564.1	urea ABC transporter permease subunit UrtB [*Aeribacillus pallidus*]	COG0559	Branched-chain amino acid ABC-type transport system, permease components
AB6A62_09150	WP_063386858.1	urea ABC transporter permease subunit UrtC [*Aeribacillus pallidus*]	COG4177	ABC-type branched-chain amino acid transport system, permease component
AB6A62_09155	WP_063386859.1	urea ABC transporter ATP-binding protein UrtD [*Aeribacillus pallidus*]	COG4674	Uncharacterized ABC-type transport system, ATPase component
AB6A62_09160	WP_066248741.1	urea ABC transporter ATP-binding subunit UrtE [*Aeribacillus pallidus*]	COG0410	ABC-type branched-chain amino acid transport systems, ATPase component
AB6A62_09165	WP_063386861.1	urease subunit gamma[*Aeribacillus pallidus*]	COG0831	Urea amidohydrolase (urease) gamma subunit
AB6A62_09170	WP_063386862.1	urease subunit beta[*Aeribacillus pallidus*]	COG0832	Urea amidohydrolase (urease) beta subunit
AB6A62_09175	WP_066248737.1	urease subunit alpha [*Aeribacillus pallidus*]	COG0804	Urea amidohydrolase (urease) alpha subunit
AB6A62_09180	WP_094245568.1	urease accessory protein UreE [*Aeribacillus pallidus*]	COG2371	Urease accessory protein UreE
AB6A62_09185	WP_094246483.1	urease accessory protein UreF [*Aeribacillus pallidus*]	COG0830	Urease accessory protein UreF
AB6A62_09190	WP_094245569.1	urease accessory protein UreG [*Aeribacillus pallidus*]	COG0378	Ni2+-binding GTPase involved in regulation of expression and maturation of urease and hydrogenase
AB6A62_09195	WP_094245570.1	urease accessory protein UreD [*Aeribacillus pallidus*]	COG0829	Urease accessory protein UreH
AB6A62_09200	WP_094245571.1	urease accessory protein UreH [*Aeribacillus pallidus*]	--	--
AMMONIUM ASSIMILATION				
AB6A62_12935	WP_130158322.1	type I glutamate--ammonia ligase[*Aeribacillus pallidus*]	COG0174	Glutamine synthetase
AB6A62_08265	TVZ77969.1	glutamine synthetase *[Aeribacillus composti]*	COG0174	Glutamine synthetase
AB6A62_03110	WP_144596851.1	FMN-binding glutamate synthase family protein *[Aeribacillus composti]*	COG0069	Glutamate synthase domain 2
AB6A62_08255	REJ15158.1	glutamate synthase [*Aeribacillus pallidus*]	--	--
AB6A62_08260	AXI39493.1	FMN-binding glutamate synthase family protein [*Aeribacillus pallidus*]	COG0069	Glutamate synthase domain 2
AB6A62_11765	AXI38791.1	NADP-specific glutamate dehydrogenase [*Aeribacillus pallidus*]	COG0334	Glutamate dehydrogenase/leucine dehydrogenase
AB6A62_12935	WP_130158322.1	type I glutamate--ammonia ligase [*Aeribacillus pallidus*]	COG0174	Glutamine synthetase
AB6A62_14885	WP_063388146.1	glutamate synthase subunit beta [*Aeribacillus pallidus*]	COG0493	NADPH-dependent glutamate synthase beta chain and related oxidoreductases

AB6A62_14890	WP_094246184.1	glutamate synthase large subunit [*Aeribacillus pallidus*]	COG0069	Glutamate synthase domain 2

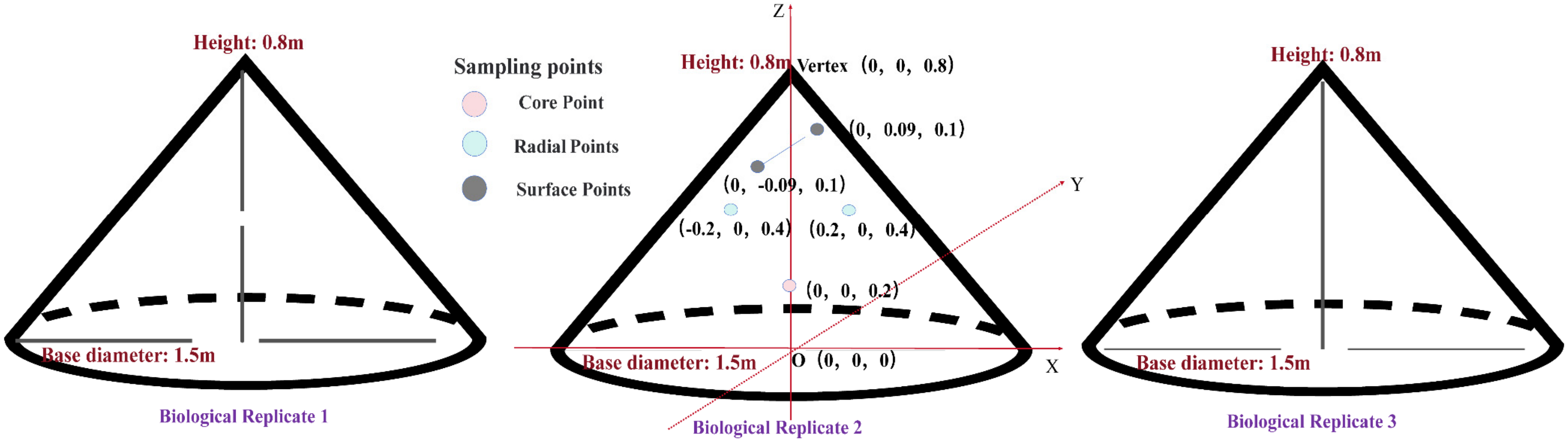

Supplementary Figure S1. Schematic of experimental unit dimensions and spatial sampling strategy. Each conical pile (1.5 m base diameter, 0.8 m height) represents one experimental unit. Three independent piles per treatment (n = 3) ensured biological replication. Sampling points are defined in Cartesian coordinates relative to the initial pile geometry (day 0): (0, 0, 0.3) core point (thermal center); (±0.2, 0, 0.4) radial points (internal transition zone); and (0, ±0.09, 0.7) surface points (outer layer). Note: Piles underwent gradual settlement over 50 days due to decomposition and moisture loss. Coordinates reflect initial geometry; sampling locations were adjusted dynamically at later time points to maintain spatial consistency.

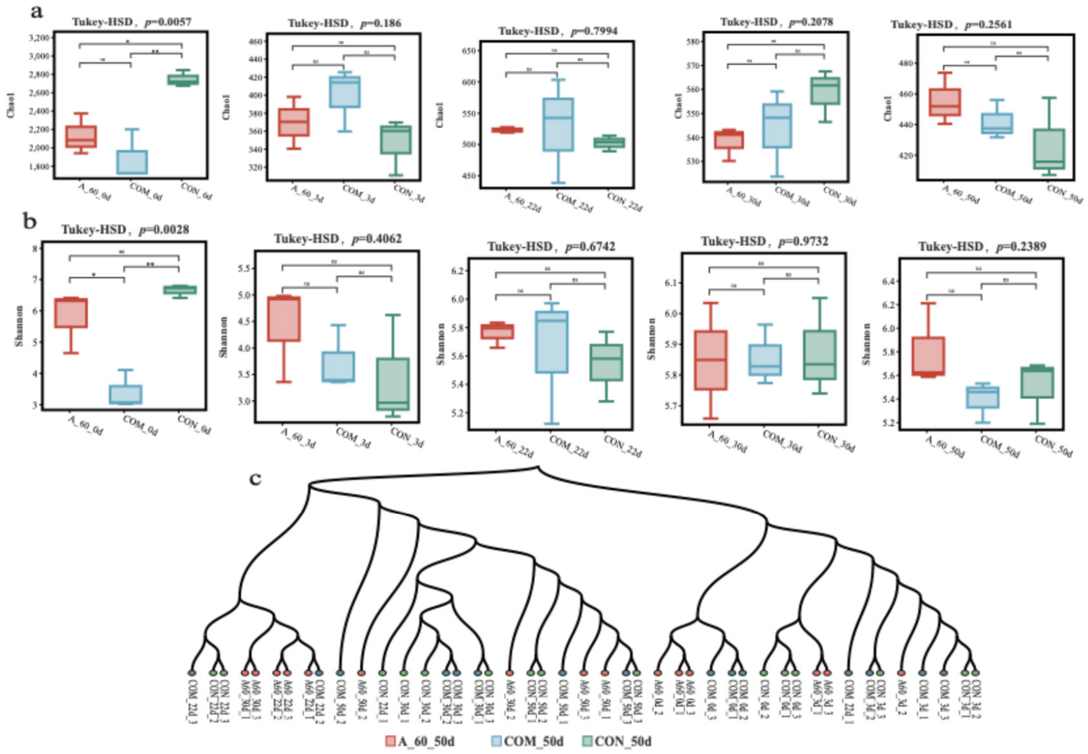

Supplementary Figure S2. Hierarchical clustering and detailed statistical comparisons of alpha diversity. (a, b) Temporal dynamics and cross-treatment comparisons of alpha diversity indices: (a) Chao1 richness estimator and (b) Shannon diversity index. Data are presented as mean ± SEM (n = 3). (c) UPGMA (Unweighted Pair Group Method with Arithmetic Mean) clustering tree based on Unweighted UniFrac distances, illustrating the structural similarity of bacterial communities across different treatments and composting stages. Samples clustering together indicate high compositional similarity. Statistical significance: Asterisks indicate statistically significant differences (* $p < 0.05$; ** $p < 0.05$; *** $p < 0.05$) between treatments (A_60, COM, CON) at the same sampling time point, while ns indicates no significant difference ($p > 0.05$), as determined by One-Way Analysis of Variance (ANOVA) followed by Tukey's HSD post-hoc test.
